# A family case report of parathyroid carcinoma associated with *CDC73* mutation in hyperparathyroidism-jaw tumor syndrome

**DOI:** 10.3389/fendo.2024.1330185

**Published:** 2024-01-29

**Authors:** Yian Gu, Yuanyuan Ye, Hua Shu, Lina Chang, Yinghui Xie, Fengao Li, Tiehong Zhu, Ming Liu, Qing He

**Affiliations:** ^1^ Department of Endocrinology and Metabolism, Tianjin Medical University General Hospital, Tianjin, China; ^2^ Department of Endocrinology and Metabolism, Baodi District People’s Hospital, Tianjin, China

**Keywords:** hereditary primary hyperparathyroidism, hyperparathyroidism-jaw tumor syndrome, parathyroid carcinoma, *CDC73* gene, parafibromin

## Abstract

**Background:**

Hereditary primary hyperparathyroidism (PHPT) accounts for 5-10% of all PHPT cases, necessitating genetic testing for diagnosis and management. Among these, hyperparathyroidism-jaw tumor syndrome (HPT-JT) is an autosomal dominant disorder caused by *CDC73* mutations with variable clinical presentations and incomplete symptoms.

**Case summary:**

The proband, diagnosed with PHPT, underwent parathyroidectomy at the age of 41 with pathological examination of parathyroid carcinoma (PC). Hereditary PHPT was initially suspected due to the early-onset PHPT and family history. Genetic testing identified a heterozygous *CDC73* mutation, NM_024529.4: c. 687_688delAG (p. Arg229Serfs*37). Even in the absence of jaw tumors, the diagnosis of HPT-JT was confirmed based on the discovery of renal cysts. A secondary thyroidectomy was performed to reduce the risk of recurrence.

**Conclusion:**

Genetic testing is strongly recommended in cases of early-onset PHPT, family history, jaw tumors, renal and uterine involvement, atypical parathyroid tumors, and PC. This testing provides valuable information for personalized management, and counseling is available for affected families.

## Introduction

Hereditary primary hyperparathyroidism (PHPT) encompasses a spectrum of syndromes that require precise classification based on glandular involvement, familial presentation, and genetic testing. One of the rare familial etiologies is hyperparathyroidism-jaw tumor syndrome (HPT-JT), which is characterized by the combination of PHPT, ossifying fibroma of the jaw, and renal and uterine disorders (1). However, the exact genotype-phenotype relationship remains to be established (2).

This paper illuminates a case of HPT-JT within a family. It is noteworthy that despite the identification of a consistent *CDC73* variant through genetic testing, the siblings presented distinct clinical phenotypes. The proband, diagnosed initially with parathyroid carcinoma (PC), underwent a second extended ipsilateral thyroidectomy to prevent recurrence. In summary, our case emphasizes the complexity of hereditary PHPT and its diverse clinical manifestations. It highlights the significance of genetic testing in guiding surgery, as it can provide essential information for personalized management.

## Case presentation

**Figure 1:**
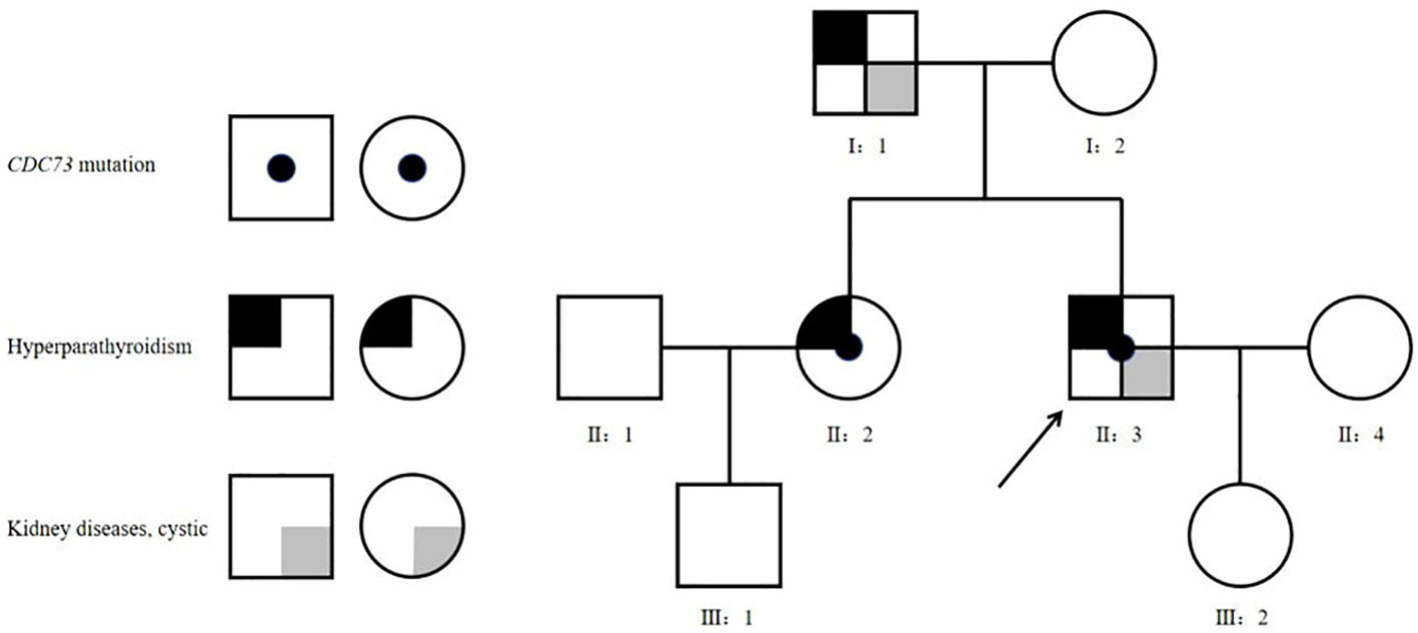
Family tree. Square - male, circle - female; arrow - the proband.

**Figure 2:**
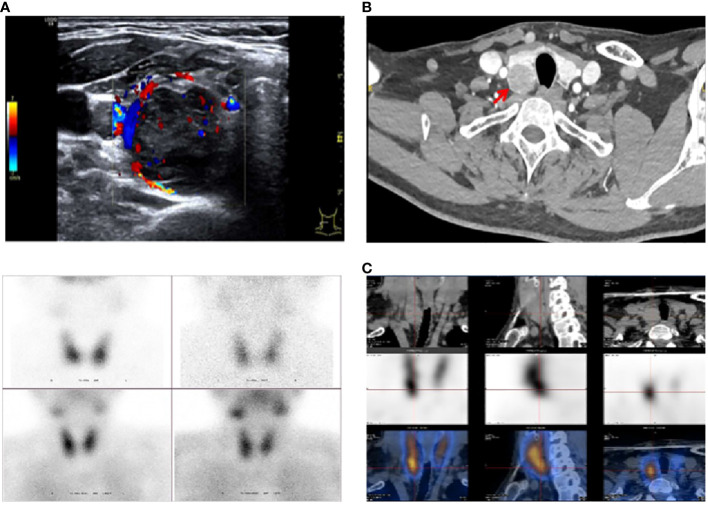
Imaging studies in the proband. **(A)** Parathyroid ultrasound: Dorsal aspect of the inferior pole of the suitable lobe: 3.1×2.4×1.9 cm. **(B)** Neck CT enhancement: Dorsal aspect of the inferior pole of the right lobe: Parathyroid origin? **(C)** Parathyroid ECT (99TcmO4¯MIBI): Right inferior parathyroid hyperfunctioning lesion: 2.2×2.3×2.9 cm.

**Figure 3:**
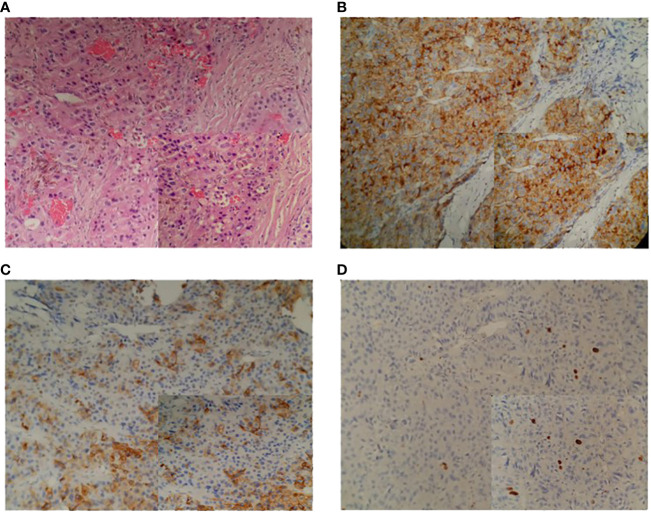
Parathyroid histopathology in the proband. **(A–D)** in order of 20x (40x in the lower right corner) HE, PTH, CgA, Ki-67 index staining.

Proband (II-3): A 41-year-old man was admitted to the hospital with a 15-year history of recurrent renal calculi and a 2-year history of a parathyroid mass. History: The patient had been experiencing recurrent renal calculi accompanied by severe abdominal pain since age 26. Importantly, he had no other symptoms such as bone pain, fractures, nausea, vomiting, thirst, or polyuria. Abdominal ultrasound revealed urolithiasis, leading to multiple extracorporeal lithotripsies. Two years ago, a parathyroid mass was incidentally discovered during a physical examination with parathyroid ultrasound, initially overlooked. Subsequently, he was referred to our hospital for a comprehensive evaluation, which encompassed the following laboratory findings: Hypercalcemia, hypophosphatemia, increased alkaline phosphatase (ALP), and elevated parathyroid hormone (PTH). Thyroid ultrasound showed a hypoechoic nodule, suspected of parathyroid origin, thus prompting his enrollment. Family history (**Table 1**, **Figure 1**): The patient’s father and sister both had parathyroid-related diseases. Physical examination revealed no evidence of thyroid goiter or renal tenderness on percussion. Admission investigations (**Table 1**): Several anomalies were noted, including hypercalciuria, vitamin D deficiency, increased bone turnover, and osteoporosis. Imaging studies (**Figure 2**), comprising parathyroid ultrasound, parathyroid ECT, and neck CT, identified a mass in the right inferior parathyroid gland. Skull radiographs indicated decreased bone mineral density without erosions or “salt and pepper” appearance. Abdominal CT disclosed bilateral renal cysts and calculi. No abnormalities were found in blood glucose, gastrin-17, adrenocortical function, plasma aldosterone-to-renin ratio, catecholamines and their metabolites, sex hormones, immunofixation electrophoresis, or pituitary MR. Surgical intervention involved the excision of the right inferior parathyroid gland. A brown mass measuring approximately 3.0 x 2.5 cm was observed, closely adherent to the adjacent thyroid tissue. Later pathology confirmed a parathyroid tumor characterized by a heterogeneous collection of neoplastic cells separated from the surrounding tissue by fibrous mesenchyme. Although there was evidence of peripheral invasion, no metastasis was observed in the nearby lymph nodes, seven of which were examined. Immunohistochemistry (**Figure 3**) revealed staining for PTH (+), CgA partial (+), CD vascular (+), Syn (-), and TTF-1 (-), with a Ki-67 index of approximately 8%.

**Table 1:** Clinical data of a family with hyperparathyroidism-jaw tumor syndrome.

History	Proband	Sister	Father
	Renal calculi: 15 years Extracorporeal lithotripsy: 6 times	Thyroid nodule: 6 years	Renal calculi: 30 years Extracorporeal lithotripsy: 3 times
Age (years)	41	38	67
Ca (mmol/L)	2.54	2.73	2.46
P (mmol/L)	0.75	0.55	0.94
U Ca (mmol/24h)	9.57	9.18	6.45
Fraction of U Ca excretion (%)	1.07	1.79	1.19
U P (mmol/24h)	40.13	25.19	31.62
ALP (U/L)	239	173	58
PTH (pg/mL)	1640.82	1753.98	107.50
25OHD (ng/mL)	4.96	11.96	10.46
OC (ng/mL)	107.90	?	27.17
β-CTX (ng/mL)	1.35	?	0.40
P1NP (ng/mL)	137.50	?	22.83
BMD	Z	Z	T
Lumbar 1-4	-2.2	-2.7	-1.5
Femoral neck	-2.6	-3.2	-2.1
Total hip	-2.9	-2.9	-1.9
Jaw Tumor	–	–	–
Renal disease	Bilateral renal cysts	–	Left renal cyst
Uterine disease	N/A	?	N/A
Pathology	Parathyroid carcinoma	Atypical parathyroid tumor	Inoperative
Pharmacotherapy	Calcitriol	Calcium carbonate, Calcitriol	Alendronate, Calcitriol
Follow-up			
Time (months)	17	42	?
Ca (mmol/L)	2.23	2.04	
P (mmol/L)	0.97	0.95	
PTH (pg/mL)	113.16	79.97	

Ca, Serum total albumin-corrected calcium, 2.15-2.55; P, Serum phosphorus, 0.8-1.45; U Ca: Urinary calcium, 2.5-7.5; U P, Urinary phosphorus, 23-48; ALP: Alkaline phosphatase, 40-150; PTH, Parathyroid hormone, 11.5-78.4; 25OHD: 25-Hydroxyvitamin D, 19.08-57.6; OC, Serum osteocalcin, 10-46; β-CTX: β-C-Terminal telopeptide region of collagen type-1, 0.31-0.7; P1NP:,Total type-1 pre-collagen amino-terminal peptide, 20-76; BMD, Bone mineral density; -, negative;?, Missing data; N/A, Not applicable.

Following the histopathological examination of the PC, immediate reoperation of the right (ipsilateral) thyroid lobe, isthmus, and paratracheal lymph nodes was performed to minimize the risk of recurrence, adhering to guidelines and clinical experience. Considering the early-onset PHPT, family history, and the rare pathology, a peripheral blood sample was sent to HUADA Medical Laboratory for whole exome sequencing. The analysis revealed a heterozygous mutation identified as *CDC73*;NM_024529.4: c.687_688delAG (p. Arg229Serfs*37), classified and documented as a pathogenic variant in accordance with the American College of Medical Genetics and Genomics standards and guidelines (3). After the two surgical interventions, the patient developed hungry bone syndrome and received calcitriol treatment.

At the one-year regular follow-up, the patient maintained normal serum calcium levels, and thyroid ultrasound exhibited no irregularities at the operative site. However, a moderately hypoechoic nodule was detected in the left thyroid lobe, categorized as TI-RADS 4 with an abnormal aspect ratio, indicating a high-grade lesion. The patient opted for close follow-up.

The table shows the clinical data of two family members: the proband’s father (I-1) and sister (II-2). The sister underwent parathyroidectomy at the age of 38. Histopathologic findings included a right lower pole parathyroid adenoma (PA) with heterogeneous hyperplasia and several small satellite nodules with aggressive biological behavior. Additionally, left upper pole parathyroid hyperplasia (PH) with a cyst was identified. Immunohistochemistry showed: PTH (+), weak Bcl-2 (+), MDM-2 (-), Cyclin D1 (+), Galectin 3 (+), CK pan (+), Ki-67 (<2%+). These results supported the diagnosis of atypical parathyroid tumor (APT). At age 44, the patient’s sister, who resides in Canada, underwent genetic testing at Genetics & Genomics, Alberta Precision Laboratories. This analysis unveiled a genetic mutation consistent with the proband. With a 30-year history of recurrent renal calculi and a recent 4-month elevation in PTH levels, the father was admitted to our department at the age of 67. Laboratory results revealed fluctuating mild hypercalcemia. Thyroid ultrasound and parathyroid CT identified a small nodule in the inferior pole of the right lobe, raising suspicion of a parathyroid origin. However, parathyroid ECT did not exhibit typical highly functional PA images. Due to the uncertainty in localization, medical intervention was initiated. At present, the patient, aged 74, refuses genetic testing.

## Discussion

PHPT is a relatively common endocrine disorder, third only to diabetes mellitus and thyroid disease in prevalence, affecting approximately 1 to 8.6 individuals per thousand. It has a higher incidence in the 45-75 age group and shows a gender bias, with women being more susceptible (4). Isolated PAs are responsible for over 80% of PHPT cases, while 0.5-5% are attributed to PCs (5). Around 5-10% of PHPT cases are hereditary. Hereditary PHPT typically manifests in late adolescence or early adulthood and follows an autosomal dominant inheritance pattern. Compared to sporadic PHPT, hereditary cases often involve multiple parathyroid glands and have a higher likelihood of recurrence. Common hereditary forms of PHPT are associated with specific genes, including the *MEN1* gene in multiple endocrine neoplasia (MEN) 1, the *RET* gene in MEN 2, the *CDKN1B* gene in MEN 4, the *CASR*, *GNA11*, and *AP2S1* genes in familial hypocalciuric hypercalcemia (FHH), the *CASR* gene in neonatal severe hyperparathyroidism, the *CDC73* gene in HPT-JT, and the *GCM2* gene in familial isolated hyperparathyroidism (FIHP) (2).

Distinguishing between FIHP and HPT-JT can be challenging as FIHP is considered either non-syndromic hereditary PHPT or an incomplete manifestation of syndromes such as MEN1, HPT-JT, and FHH associated with *MEN1*, *CDC73*, and *CASR* mutations (6). Latest literature also links *GCM2* mutations to FIHP, necessitating further investigation (4).

HPT-JT, a rare familial form of hereditary PHPT with an incidence of less than one in a million, is attributed to mutations in the tumor suppressor gene *CDC73* (formerly *HRPT2*), identified in 50-80% of cases (1, 2). The prevalence tends to rise with age, though onset may occur as early as age 7 (1, 5). Clinical manifestations are variable and incomplete. Unlike other hereditary forms of PHPT, HPT-JT is typically associated with involvement of a single parathyroid gland (7). PHPT is reported in 80-90% of HPT-JT cases, and there is a high incidence of PC, with a risk as high as 15-20% (8). In a national retrospective study of *CDC73*-associated PHPT in the Netherlands, eleven (12.4%) pathogenic germline mutations were identified in 89 patients with clinically heterogeneous PHPT. The mean age ( ± SD) at diagnosis was 32 ( ± 15) years with a range of 13-54 years (9). The early onset of clinical manifestations and the variable pathology in this family highlight the complexity of this disease. Despite its name, only about 30-40% of patients have maxilla and/or mandible fibro-osseous tumors, which are classified by the World Health Organization as slow-growing, painless, benign growths with a malignancy risk of less than 0.5% (10). They are primarily driven by genetic mutations rather than hyperparathyroidism (10, 11). Importantly, PHPT-induced fibrous dysplasia (osteoid osteoma) tends to resolve spontaneously after parathyroidectomy. Composed of fibroblastic mesenchyme and minerals, fibro-osseous tumors have distinct morphologic features that persist after parathyroidectomy (10). Treatment should be based on the size, location, and biological characteristics of the tumor, with postoperative surveillance recommended to prevent recurrence. Renal involvement constitutes 15% of HPT-JT cases, including cystic disease, hamartoma, and carcinoma, of which cystic disease is the most prevalent (12). These changes can manifest as small cysts or even polycystic kidney disease, either alone or in combination with rare tumors, requiring renal replacement therapy in severe cases (9, 12). Both the proband and his father were found to have simple renal cysts with normal renal function. Uterine disorders, such as leiomyoma, adenomyosis, endometrial hyperplasia, and sarcoma, are the second most common manifestations, affecting over half of women (6, 7). Although there is no information on the proband’s sister, regular follow-up is recommended. While other cancers have occasionally been reported in HPT-JT patients, the relationship to the syndrome remains uncertain. Vigilance is crucial for potential cancers, especially involving the thyroid, pancreas, colon, and testes (1, 2, 9). Following a year after surgery, the proband exhibited a TI-RADS 4 thyroid nodule, which carries a 9.1% risk of thyroid cancer (13). Fine-needle aspiration biopsy is advised if necessary. The differentiation of hereditary PHPT depends on the clinical presentation. However, the renal and uterine disorders are frequent manifestations that complicate the definitive diagnosis, especially when they coexist with PHPT. Furthermore, even in cases of isolated PHPT, HPT-JT should be considered. Therefore, genetic testing is required for diagnosis.

The *CDC73* gene on chromosome 1q31.2 consists of 17 exons and encodes parafibromin, a 531-amino acid protein with antiproliferative properties (14). Parafibromin is expressed in various tissues, including parathyroid, adrenal, kidney, heart, and skeletal muscle. It primarily localizes within the nucleus, where it controls cell proliferation, apoptosis, and maintains chromosome stability as part of the human PAF1/RNA polymerase II-related complex (15). The pathogenesis of HPT-JT aligns with the two-hit hypothesis proposed by Knudson, in which a heterozygous germline mutation (inherited from a mutant parent or acquired during embryonic development in rare cases) is coupled with a somatic alteration (16). These lead to the loss of both alleles, resulting in the loss of heterozygosity within the tumor DNA. Consequently, the absence of parafibromin expression contributes to the development of neoplasia (15, 16). Genetic variations are distributed throughout the coding region and splice site, predominantly concentrated in exons 1, 2, and 7. Mutations in exons 3 and 4 are infrequent, while large intragenic deletions and partial intronic mutations are rare (8). The majority of *CDC73* mutations, more than 75%, are frameshift and nonsense mutations (17). Although a clear genotype-phenotype relationship has not been formalized, recent research suggests that HPT-JT cases with frameshift mutations, nonsense mutations, and large intragenic deletions have an almost 7-fold higher risk of PC than missense mutations (8). In this family, a two-nucleotide deletion in exon seven of the *CDC73* gene was identified, which was predicted to cause a frameshift and the premature termination codon, resulting in nonsense-mediated decay or a truncated protein. This exon variant has been identified in a Dutch family and a Chinese patient, but the clinical presentation differed between the index cases (9, 18).

Genetic testing plays a critical role in confirming hereditary PHPT, detecting associated complications, and providing essential guidance for surgical intervention. It is appropriate for individuals with early-onset PHPT, jaw tumors, renal and uterine disorders, APT, and PC. As other family members may be at risk of carrying variants, genetic testing of offspring may facilitate early tumor detection. In this family, a consistent mutation in the *CDC73* gene was identified through national and international genetic testing. Unfortunately, other high-risk relatives did not undergo validation, missing an opportunity for early intervention and risk assessment.

PC is an extremely rare malignant endocrine tumor, comprising only 0.005% of all cancers (5). It usually presents as a sporadic tumor, but occasionally occurs in individuals with hereditary PHPT, leading to life-threatening hypercalcemia due to excessive production of PTH (5, 19). Preoperative serum calcium exceeding 3 mmol/L with PTH over three times the upper limit of normal and parathyroid lesions larger than 3 cm, referred to as the “>3+>3+>3 principle”, strongly suggests the possibility of PC (5, 8, 20). Nevertheless, histopathologic tissue biopsy remains the gold standard. The proband conforms to the above principle but also has severe 25-hydroxyvitamin D (25OHD) deficiency. Concerning secondary hyperparathyroidism, the expected upper limit of normal PTH (PTHmax, pg/mL) can be calculated using the formula 120−[6×serum calcium(mg/dL)]−[0.5×25OHD(ng/mL)]+[0.25×age(years)] , as shown in Jin’s diagnostic alignment diagram for PHPT with vitamin D deficiency (21). In this family, the level of PTH was significantly higher than PTHmax, which is consistent with a diagnosis of PHPT. It is noteworthy that avitaminosis D is prevalent in PHPT with potential mechanisms including increased conversion of 25OHD to 1,25-dihydroxyvitamin D and accelerated metabolism of 25OHD (21, 22). The patient underwent surgery without vitamin D supplementation due to strong motivation, family history of PHPT, kidney stones, and osteoporosis. Unfortunately, most cases of PC are diagnosed postoperatively. Fine-needle aspiration biopsy carries the risk of tumor dissemination and misdiagnosis because cytomorphology does not adequately differentiate between benign and malignant entities (5). Even experienced pathologists find it challenging to differentiate PC from APT. The diagnosis of PC relies on identifying vascular, lymphatic, perineural invasion, infiltration of adjacent structures, or histologic/cytologic metastasis (5, 19). However, APT may exhibit similar histologic features, such as adhesion to nearby structures, monotonous sheet-like or trabecular growth, fibrosis, necrosis, increased mitotic activity, cytologic atypia, and cellular extension into the capsule without complete penetration (19, 23). Immunohistochemistry can improve the accuracy of diagnosis, with positive staining for PTH and CgA often indicating parathyroid tissue (23). Additionally, a Ki-67 index greater than 5% suggests PC (5). The *CDC73* mutation, resulting in the deletion of parafibromin, is found in approximately 70% of sporadic PC cases and only about 2% of PA cases, indicating a high risk of recurrence, metastasis, and mortality (23). Loss of parafibromin is emerging as a more promising predictor of adverse outcomes than mutation (5, 20). Therapeutically, surgery is a crucial prognostic determinant, with limited effects of radiotherapy and chemotherapy. Extensive surgery is advocated for PC, including complete resection of the primary tumor, ipsilateral thyroid lobe and isthmus, adjacent structures, and metastatic lymph nodes, to reduce the risk of persistence or recurrence (20, 24). Studies have shown that patients with PC who do not receive a complete resection at the initial operation may benefit from removal of the ipsilateral thyroid lobe and central group lymph nodes at reoperation within one month (24). The management of HPT-JT is based on the European Society of Endocrine Surgery guideline, which recommends selective resection of the abnormal parathyroid gland in cases of single involvement (1, 6). In instances where preoperative localization is negative or equivocal, bilateral neck exploration plus subtotal parathyroidectomy is preferred (6, 7, 25). Prophylactic parathyroidectomy increases the risk of postoperative hypoparathyroidism. A decline of more than 50% in intraoperative PTH indicates a successful procedure (23). In our case, both the proband and his sister experienced a significant decrease in PTH, demonstrating effective removal of the parathyroid mass causing PHPT. Furthermore, the overall survival rate for PC is 78-85% at 5 years and 49-70% at 10 years (5, 6).

Although there are no formal guidelines for the management of HPT-JT, individuals carrying the mutation should undergo lifelong follow-up. Surveillance should include regular monitoring of serum calcium, serum phosphorus, and PTH every six months to one year starting at 5-10 years of age. In addition, screening with parathyroid, renal, and gynecologic ultrasound, as well as jaw radiographs, should be conducted every five years beginning at 10 years of age (1, 2, 12). Genetic counseling is available to affected families.

In conclusion, this case report details the clinical features, pathological observations, and genotypic profiles within a family affected by HPT-JT, highlighting the variability in clinical manifestations and the tendency towards incomplete symptoms. Our primary objective is to raise awareness among clinicians about the significance of genetic testing and counseling in guiding surgical interventions and establishing routine follow-up.

## Data availability statement

The original contributions presented in the study are included in the article/supplementary material, further inquiries can be directed to the corresponding author/s.

## Ethics statement

The requirement of ethical approval was waived by the Medical Research Ethics Committee of Tianjin Medical University General Hospital for the studies involving humans. The studies were conducted in accordance with the local legislation and institutional requirements. The participants provided their written informed consent to participate in this study. Written informed consent was obtained from the individual(s) for the publication of any potentially identifiable images or data included in this article.

## Author contributions

YG: Data curation, Writing – original draft, Writing – review & editing. YY: Data curation, Writing – review & editing. HS: Formal analysis, Writing – review & editing. LC: Writing – review & editing. YX: Writing – review & editing. FL: Writing – review & editing. TZ: Writing – review & editing. QH: Supervision, Writing – review & editing. ML: Supervision, Writing – review & editing.
